# Atomic-Scale Structural Fluctuations of a Plasmonic Cavity

**DOI:** 10.1021/acs.nanolett.1c02207

**Published:** 2021-08-24

**Authors:** Anna Rosławska, Pablo Merino, Abhishek Grewal, Christopher C. Leon, Klaus Kuhnke, Klaus Kern

**Affiliations:** † Max-Planck-Institut für Festkörperforschung, D-70569 Stuttgart, Germany; ‡ Université de Strasbourg, CNRS, IPCMS, UMR 7504, F-67000 Strasbourg, France; § Instituto de Ciencia de Materiales de Madrid, CSIC, E-28049 Madrid, Spain; ∥ Instituto de Física Fundamental, CSIC, E-28006 Madrid, Spain; ⊥ Institut de Physique, École Polytechnique Fédérale de Lausanne, CH-1015 Lausanne, Switzerland

**Keywords:** plasmonics, picocavity, atomic-scale structure, point contacts

## Abstract

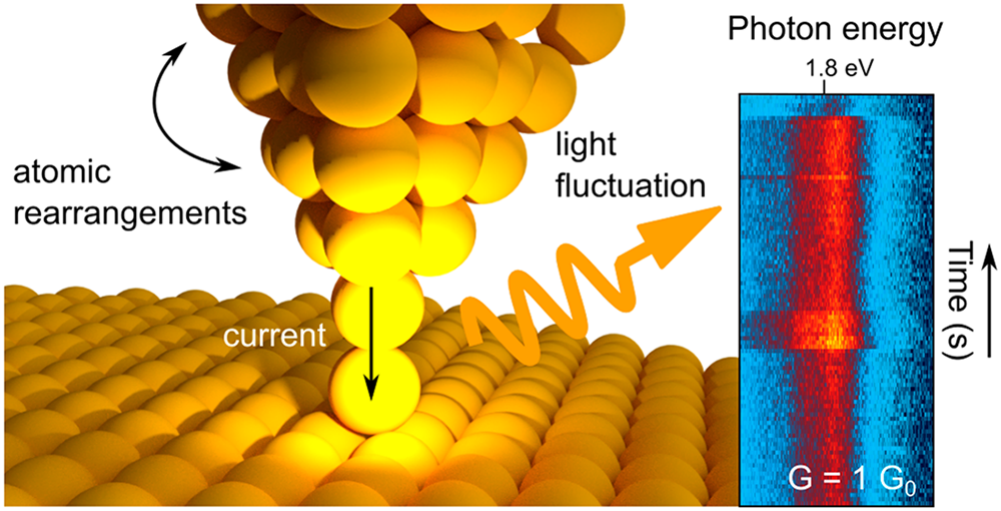

Optical spectromicroscopies, which can reach atomic resolution due to plasmonic enhancement, are perturbed by spontaneous intensity modifications. Here, we study such fluctuations in plasmonic electroluminescence at the single-atom limit profiting from the precision of a low-temperature scanning tunneling microscope. First, we investigate the influence of a controlled single-atom transfer from the tip to the sample on the plasmonic properties of the junction. Next, we form a well-defined atomic contact of several quanta of conductance. In contact, we observe changes of the electroluminescence intensity that can be assigned to spontaneous modifications of electronic conductance, plasmonic excitation, and optical antenna properties all originating from minute atomic rearrangements at or near the contact. Our observations are relevant for the understanding of processes leading to spontaneous intensity variations in plasmon-enhanced atomic-scale spectroscopies such as intensity blinking in picocavities.

Modern nanoscale spectroscopies routinely reach single-molecule sensitivity^1,2^ and are even capable of achieving contrast within individual molecules.^3−5^ These methods, such as surface-enhanced Raman spectroscopy (SERS),^6,7^ tip-enhanced Raman spectroscopy (TERS),^1,3,4,6,8,9^ tip-enhanced photoluminescence (TEPL),^10^ and scanning-tunneling-microscopy-induced luminescence (STML),^2,5,11−17^ owe their excellent resolution to the local enhancement of the electromagnetic field at a local hotspot of a metallic film, a nanoparticle, or a sharp tip, further boosted by the tunneling current localization in STML. Such enhancement originates from the lightning rod effect amplified by collective oscillations of charges, i.e., plasmons. Both phenomena bolster the coupling of local electromagnetic fields to the far-field, which results in an increased signal detected at a macroscopic distance from the investigated structure. Because the cavity geometry is intrinsically of atomic scale, the atomic arrangement and stability of the plasmonic antenna can play a crucial role in the enhancement mechanism. In the extreme case, the electromagnetic field is confined at the scale of a single atom, constituting a so-called picocavity,^18^ which enables addressing optical signals with submolecular resolution.^3,10,13,19−21^ The role of the atomic structure becomes apparent also in the blinking signal attributed to atomic-scale fluctuations of the plasmonic cavity^6,18,22^ irrespective of additional chemical or adsorption site modifications of the investigated system. The significance of both the static and fluctuating atomic structure on plasmonic properties has been studied both theoretically^23−32^ and experimentally,^10,22,33−36^ the latter, however, lacking precise characterization at the single-atom level.

Here, we address this issue using the well-controlled atomic-scale environment in a low-temperature scanning tunneling microscope (STM) in ultrahigh vacuum (UHV). We build the plasmonic structures of interest by depositing single Au atoms from a Au tip on a clean Au(111) surface and approaching them again with the tip until a single-atom contact is formed. Applying a voltage bias across such a contact results in electroluminescence due to the decay of the plasmonic modes excited in the junction by the current. The resulting light emission signal can be temporally monitored.^12,14,37−41^ We observe irreversible changes in the plasmonic properties of the junction as well as light intensity fluctuations on the temporal scale of seconds, both of which are correlated with the transport properties of a single-atom contact. Our results agree with theoretical predictions^23,28^ and show that minute changes in the atomic structure at or near the junction substantially modify the properties of the plasmonic antenna.

The experiments have been performed in a home-built low-temperature (4 K) UHV STM with optical access.^42^ We couple its outputs to a single-photon avalanche photodiode (SPAD, MPD-PDM) and an optical spectrograph (Acton SP 300i, CCD camera: PI-MAX 4). The spectra presented in this work have not been corrected for the detector sensitivity. The Au(111) crystal is cleaned by repeated cycles of Ar^+^ sputtering and annealing (up to 850 K). We used electrochemically etched^43^ Au wires for tips.

First, we study the influence of a single-atom transfer on the plasmonic properties of the junction. Figure 1(a) shows an STM image recorded from top to bottom in constant current mode. During acquisition, the scanning is interrupted at the position marked by the arrow, where a single atom is deposited by closely approaching the surface with the tip (see the Supporting Information for procedural details).^10,44^ The scan is then continued as before. The spectrally integrated light intensity is simultaneously recorded during the scan, Figure 1(b), revealing that the deposition of an atom changes the observed photon yield. In the lower part of Figure 1(b), the intensity averaged over an area of homogeneous electroluminescence is 22% lower than in the upper part for the same tunnel current. Over several measurements with different tips, we find that the electroluminescence changes in the range of 3–22%. However, the shape of the spectra obtained before and after atom deposition (Figure 1(c)) are similar, suggesting that the excited plasmonic modes in the STM junction do not change significantly. Spatially, we observe a reduced electroluminescence intensity (Figure 1(b)) on top of the deposited atom and on a surface defect (marked by a dashed circle), which can be assigned to the local variation of the density of electronic states of the sample that modulates the electroluminescence.^15,16^ However, the overall reduction of the plasmonic signal can be interpreted as a change of a local cavity structure due to a single-atom transfer, which affected both the plasmonic enhancement and the local density of states of the tip.

**Figure 1:**
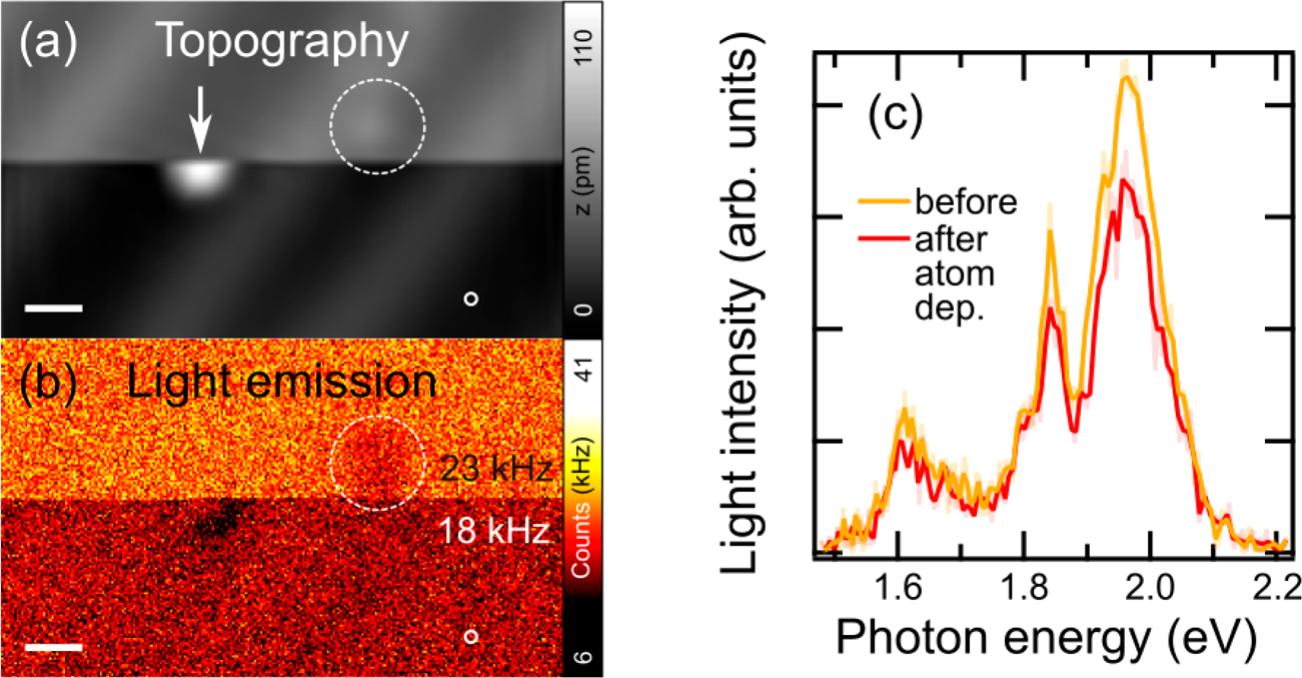
(a) STM topographic image of the Au(111) surface recorded under electron tunneling conditions, *U* = −2.5 V, *I* = 1 nA. During the scan (from top to bottom), a single atom was deliberately deposited from the tip apex onto the surface by atomic manipulation at the position marked by the arrow (for details, see text). Scale bar: 1 nm. (b) Light intensity map recorded simultaneously with (a). The values in the bottom and upper part of the image indicate the average light intensity before and after tip modification. (c) Optical spectra recorded on the position marked by the small circles at the bottom right of (a) and (b) before (yellow curve) and after (red curve) atom deposition; *U* = −2.5 V, *I* = 1 nA, integration time: 50 s.

Next, we employ atomic contact experiments to increase the sensitivity for tip apex changes (Figure 2(a)). Generally, single-atom contacts manifest their quantum nature through conductance (*G*) quantization. For each fully open current transmission channel, the conductance is 1 *G*_0_ = 77.48 μS, as derived in quantum transport.^45^ In the case of Au, a conductance of 1 *G*_0_ indicates that the contact is formed between two atoms only and the conductance is dominated by one transmission channel.^46^ To reduce heat dissipation at high currents in our experiment, we apply a rather low voltage (on the order of 1 V) and operate the junction in an overbias light emission regime that leads to geometrically more stable junctions that undergo changes only on a time scale of seconds and remain intact over extended measurement times. Overbias emission occurs when the energy of the emitted photons exceeds the potential difference *U* seen by the transmitted electrons, *h*ν > *eU*.^47−53^ The underlying mechanism is strongly debated in the literature and has either been assigned to plasmon-mediated coherent interaction between electrons^50,54−56^ or photon emission from a hot electron gas.^49,51,57^

**Figure 2:**
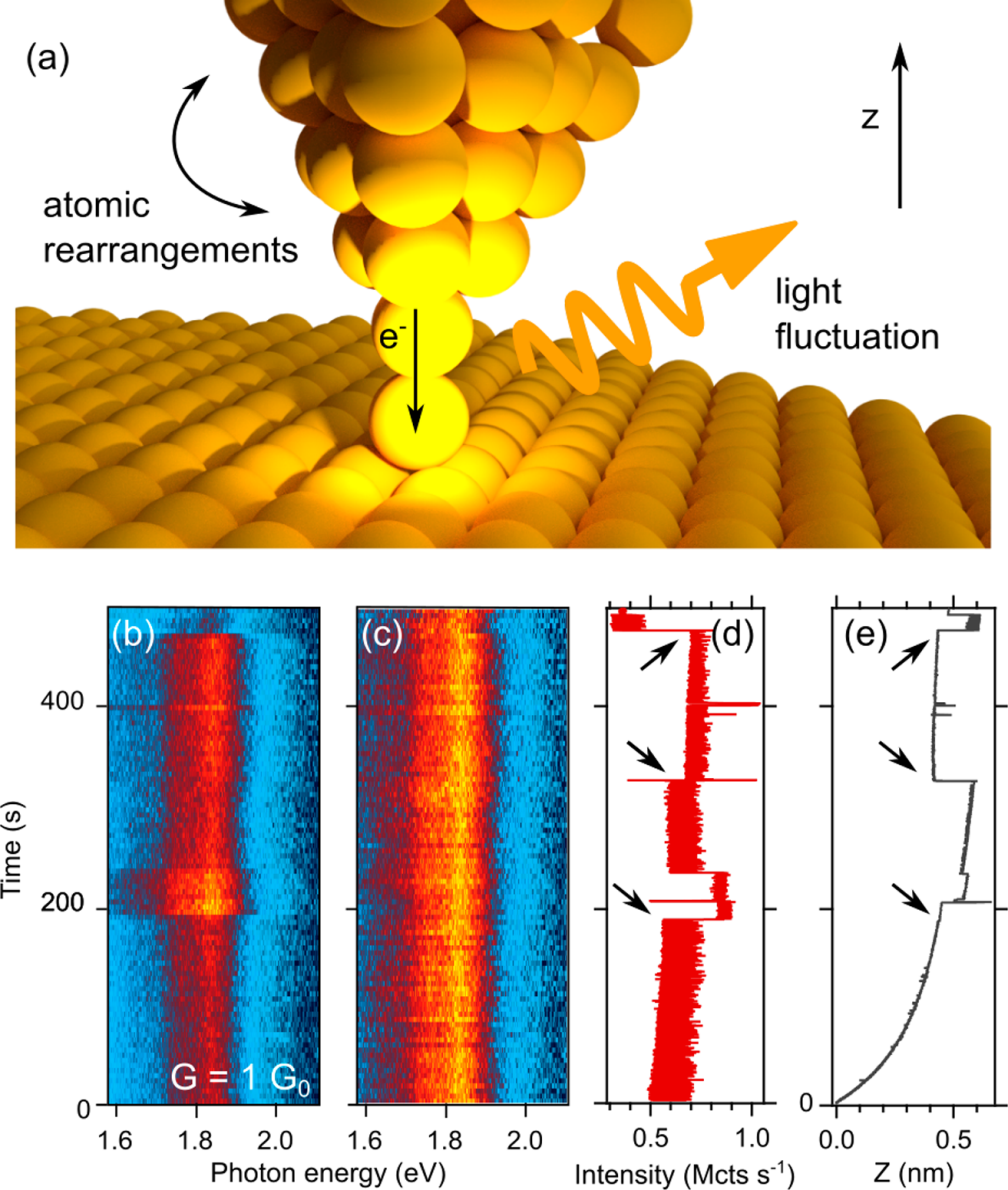
(a) Illustration of the experiment in which the tip of an STM forms a single-gold-atom contact. The current passing through the junction excites the luminescence. During measurements, the current, position, and light emission are monitored. (b) Time-trace of optical overbias emission spectra measured for a single-atom contact with a conductance of 1 *G*_0_. The plot consists of 100 spectra, each recorded with 5 s of integration time. (c) Spectra from (b) normalized to the maximum. (d,e) Simultaneously recorded light intensity measured by the SPAD (d) and *z* position (e) with a 20 ms integration time per point. The current feedback was enabled during the measurement to maintain 1 *G*_0_, *U* = 1 V, *I* = 77.48 μA.

The stability at low bias condition, supported by a low-temperature (4 K) environment, enables maintaining a single-atom contact for several minutes using the constant current feedback loop of an STM. After a single-atom contact has been formed, we turn on the feedback loop to stabilize the conductance at 1 *G*_0_ by adjusting the tip height *z*. Simultaneously, we monitor the optical spectrum (Figure 2(b,c)), the integrated light intensity measured by the SPAD (Figure 2(d)), and the relative change of the *z* position (Figure 2(e)) as a function of time. During the measurement, we observe electroluminescence intensity variations, while the shape of the normalized spectra (Figure 2(c)) remains unchanged within our experimental precision. This is remarkable, since even minor voltage pulses of only a few milliseconds can modify tips enough to affect the spectral shape substantially. Such pulses can shift plasmon lines by tenths of an eV and usually affect the relative intensities of different spectral modes by modifying the mesoscopic tip structure,^58−61^ but these changes are not seen here. As observed, the constant current and constant voltage condition is unable to induce such strong tip modifications.

Upon starting the measurement of Figure 2, one can immediately observe a tip retraction of more than 0.5 nm (Figure 2(e)), due to a tip elongation while the feedback loop is set to keep a constant current (77.48 μA, corresponding to 1 *G*_0_) condition. The tip elongation can be linked to thermal expansion due to power dissipation (see Supporting Information). In our study, we use bias voltages on the order of 1 V, which are necessary to drive the light emission, in contrast to bias voltages in the range of a few mV usually applied to study atomic contacts for which power dissipation effects are negligible. Remarkably, a measurement in contact results in only minor modifications to the surface, reflecting the fact that it is mostly the structure of the tip that is altered (see Supporting Information for more details).

In addition to a continuous relaxation of geometry, rapid steps of the emission intensity can also be identified even though the light emission in Figure 2 is driven by a constant current. With respect to the latter, we find three different types of behavior: At *t* = 195 s, an extremely small alteration of geometry (Δ*z* = 6 pm) is accompanied by a significant rise of intensity (+40%), at *t* = 335 s, a major decrease of *z* (Δ*z* = −170 pm) leads to a small increase of intensity (+8%), and at *t* = 470 s, a large increase of *z* (Δ*z* = 180 pm) leads to a drastic reduction of emission (−47%). These events are marked by arrows in Figure 2(d,e). Every such irreversible intensity modification is assigned to a geometry change at the tip or, more generally, in the junction; however, their relative size and even relative sign seem to be completely arbitrary. We also observe that fluctuations of the current due to reversible junction instabilities (noise in Figure 2) counteracted by the feedback loop translate into reversible fluctuations in the light emission. Additionally, we observe that the intensity modifications do not show any significant emission angle dependence, as evidenced in Figure 2(b,d), where these changes are of the same order and sign while the light is recorded in two different emission directions by two independent detectors.^42^ The results of Figures 1 and 2 together show that single-atom changes to a tip apex and its repositioning on the order of a single-atom length do not modify the spectral envelope of the electroluminescence but do vary its intensity drastically.

Monitoring the geometry changes without the interference of a feedback circuit allows us to probe the relation between the optical and transport properties in more detail. We follow the rupture process of the junction by retracting the tip at a constant speed of 5 pm/s while simultaneously monitoring light intensity and conductance (Figure 3). In contrast to the experiment in Figure 2, here the junction is deliberately put under increasing mechanical tension that provokes successive modifications to the junction. As expected from such a break-junction experiment,^45^ we observe steps in the conductance that are often close to multiples of *G*_0_, indicating that our junction consists of more than one atom. Note that the total displacements in Figure 3(a–c) are only 200 to 400 pm, which is no more than 1 to 2 times the nearest neighbor distance of two Au atoms. Apart from large conductance steps, which can be attributed to a reduction of transport channels resulting from the removal of atoms from the narrowest constriction, fractional steps in the conductance are also observed. Again, conductance and light intensity changes occur together, but no clear correlation in relative magnitude and sign can be determined. Under these experimental conditions, minimal changes of conductance can have strong consequences for emission intensity (Figure 3(b)). The optical spectra recorded at high conductance values (see the Supporting Information) reveal the expected and reversible blue-shift of the plasmonic modes due to the modifications in local charge density distribution.^23,28,62^ However, the result remains broadly consistent with changes at the very tip apex being primarily expressed in the electroluminescence intensity and not the optical spectral envelope. These observations point at the occurrence of a number of different processes.

**Figure 3:**
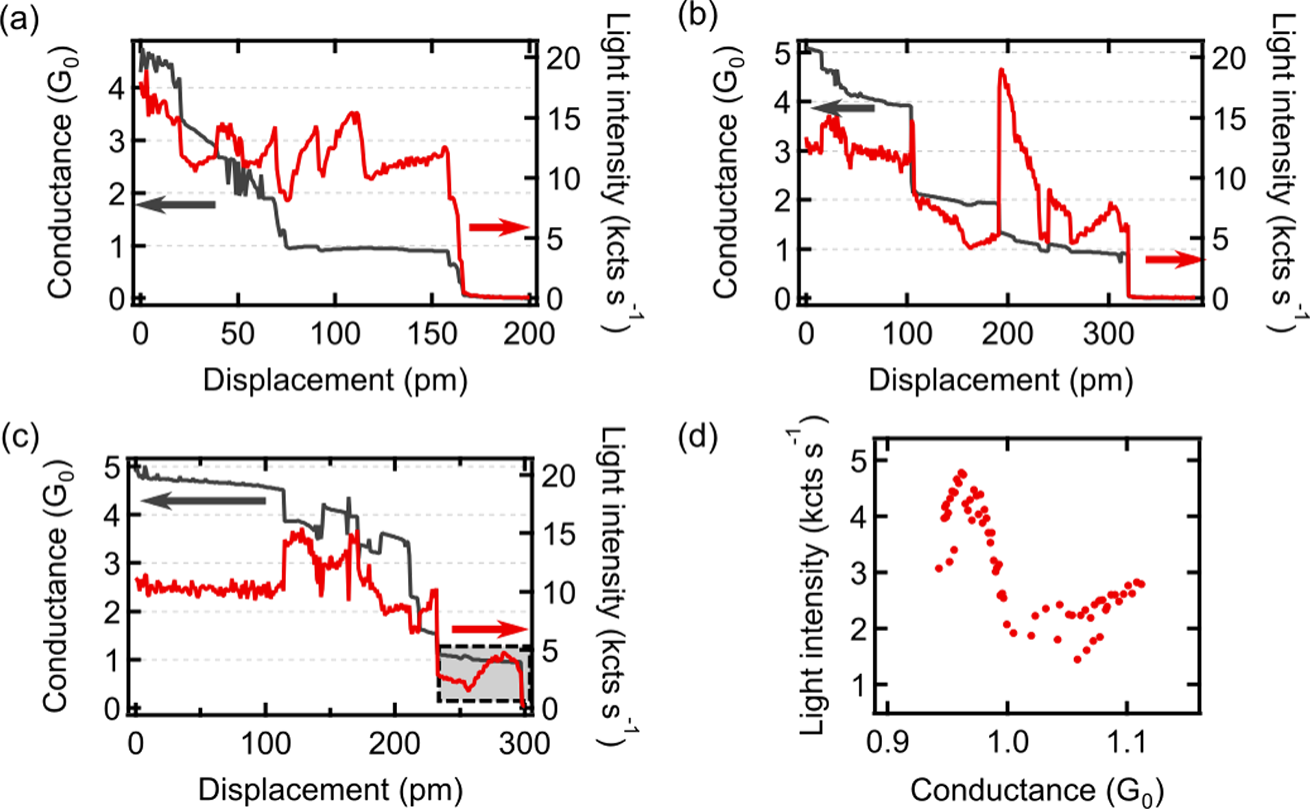
(a–c) Conductance (dark gray) and overbias light intensity (red) recorded during tip retraction with a constant speed of 5 pm s^–1^. The three different point contacts in (a–c) were formed by approaching the tip toward the same surface area until a conductance of 5 *G*_0_ was reached. *U* = −0.8 V. (d) Light intensity vs conductance curve extracted from the data marked by the gray rectangle in (c). Note the existence of a local minimum at 1 *G*_0_. Further data sets can be found in the Supporting Information.

Our results show that a junction operated under steady-state or dynamic conditions will incur electrical, mechanical, and thermal fluctuations that modify its electroluminescent properties. In general, the rate of light emission (*R*) excited by the current in an STM junction can be expressed as^50,63^
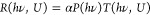
1with *P*(*h*ν) being the spectral plasmonic enhancement factor depending on the optical density of states,^63^*T*(*h*ν, *U*) dictating the energy- and bias-dependent charge transport, which excite the plasmonic modes in the junction, and α being an experimental scaling parameter. Both *P*(*h*ν) and *T*(*h*ν, *U*) depend on the geometry of the junction, which affects the spectral shape through the local plasmonic density of states and electronic transport, respectively.

First, we consider the effect of the current on the emission. At higher currents, more photons can be emitted by increasing *T*(*h*ν, *U*). Upon decreasing conductance, we expect a more than proportional reduction of light emission due to the higher-order overbias emission mechanism. Examples can be seen in Figure 3(a) at *z* = 25 pm, *z* = 70 pm, and *z* = 170 pm, where a conductance decrease leads to a decrease in emission. Focusing on the intensity behavior around 1 *G*_0_ conductance (Figure 3(d)), we find an emission minimum due to shot noise minimization at integer multiples of *G*_0_, which has been shown to result in a minimum of light emission.^48^ Such minima are often obscured in the experiment, because more than one conduction channel may be involved in the charge transport.

Next, we consider the apical atoms that form the tip antenna and their reorganization due to mechanical rupture-induced processes. Comparing Figure 3(a) and 3(b) suggests that while in (a) conductance steps reduce emission, but the slow relaxation tends preferentially toward higher values of light intensity, in (b) the tip jumps to higher emission but relaxes mostly toward lower values of light intensity. Similarly to the discussion of the steps in Figure 2, also for the relaxation processes in Figure 3, there appears to be no preferred sign of change in emission when the conductance reduces or increases. Conductance changes can be related to modifications in the atomic structure of the junction.^23,45,46,51^ Variations on the order of 1 *G*_0_ can be assigned to a removal/addition of single atoms at the narrowest constriction.^45,64^ Finer changes may occur due to atomic rearrangements near the junction, such as an atom at the tip moving while breaking and forming bonds with its neighbors.^23,46^ Rearrangements influence the transmission coefficients of the channels involved in the transport (*T*(*h*ν, *U*)),^45^ and as a consequence, the luminescence changes. Similar effects can occur when the tip is expanding thermally, as in Figure 2(d,e). In addition to mechanical rupture due to either tip displacement or thermal effects, the atomic structure may spontaneously rearrange as a result of the current flow that induces electromigration,^65^ which is efficient for gold and has often been employed to fabricate single-atom junctions.^51^

The changes in the plasmonic properties of the junction described by *P*(*h*ν) in eq 1 are also related to the modifications of atomic structure,^18,23,24,26−30,66^ and are observed in photoluminescence,^10^ that is, without current excitation. In particular, Rossi^23^ et al. and Marchesin^28^ et al. calculated that in point contacts the plasmonic mode intensity is sensitive to the atomic-scale structure and the modifications of the modes coincide with changes in the conductance with the sign depending on the mode. Our results agree remarkably well with these theoretical predictions regarding the total emitted intensity and confirm the occurrence of simultaneous conductance and luminescence steps. The impact on the charge flow in a slightly modified atomic structure, regardless of the plasmon excitation *T*(*h*ν, *U*) results in the local modification of polarizability that is reflected in the intensity of the plasmonic response *P*(*h*ν). In our experiments, we monitor a higher-order overbias emission that depends on higher powers of both *P*(*h*ν) and *T*(*h*ν, *U*),^50^ and thus, even a subtle change in the atomic structure may result in a substantial modification of the luminescence intensity. This explains the significant changes in the light intensity (as at *z* = 190 pm in Figure 3(b)) observed in our experiments and demonstrates that overbias electroluminescence is an intrinsically sensitive probe to changes of the atomic structure in point contacts. Indeed, occasionally, a step change in the light intensity occurs with an insignificant change in the conductance as in Figure 3(a) at *z* = 115 pm or *z* displacement (Figure 2(c,d), *t* = 195 s), which can be attributed to an atomic rearrangement further away from the narrowest constriction.

In summary, we have investigated and characterized spontaneous variations of plasmonic luminescence output in response to different current or tip displacement control parameters at the single-atom limit. This study was carried out for electroluminescence from Au single-atom contacts in UHV at cryogenic temperature in an exceptionally well-defined environment, profiting from the precision achievable by STM. We can ascribe the observed fluctuations to current changes as well as to mechanical stress and thermal effects that modify the atomic structure of the tip apex and reproduce key theoretical predictions correlating modifications of conductance with modifications of plasmonic properties,^23,28^ both originating from the modified electronic structure. Even under well-controlled conditions, we observe a large variety of modifications of the intrinsic plasmonic response of the system *P*(*h*ν) and the plasmon excitation efficiency *T*(*h*ν, *U*), which call for detailed calculations on their atomistic mechanisms. Our results have stark implications in spectroscopies other than STML that leverage plasmonic enhancement such as SERS, TEPL, and TERS. These atomic-scale phenomena are in particular critical for picocavities^10,13,18−20^ and measurements in the contact regime^9,47,67^ above cryogenic temperatures or when significant currents are employed. When local optical enhancement at the junction is considered, single-atom manipulation and stabilization of the junction may be employed to stabilize the output signal,^10^ facilitating studies with submolecular resolution. We observe the effects of slightly elevated temperature (ca. 50 K). At higher temperatures (e.g., room temperature) or under laser illumination, spontaneous atomic rearrangements are even more probable and thus result in undesired intensity blinking that would modulate the desired optical signal during investigations of more complicated systems. Such fluctuations may also affect the efficiency and stability of optical antenna devices based on the emission of plasmonic light excited by inelastic tunneling.^51,68−71^ In this respect, further investigations and development of stabilization strategies will be of significant interest.

## Supporting Information Available

Single-atom deposition, measurements with the feedback loop closed, luminescence fluctuations in a point contact to a single atom deposited on the surface, local heat dissipation in the junction, optical spectroscopy at high conductances, emission minimum around integer *G*_0_ (PDF)
