# Supplementary material for: Atomic-Scale Structural Fluctuations of a Plasmonic Cavity
Source: Nano Lett. 2021 Aug 24;21(17):7221–7. doi: 10.1021/acs.nanolett.1c02207 (PMC8887667; doi:10.1021/acs.nanolett.1c02207)
Supplement: Supplementary file 1 — nl1c02207_si_001.pdf [file nl1c02207_si_001.pdf]

Supporting Information for:

Atomic-scale structural fluctuations of a plasmonic cavity

Anna Rosławska[1,2,*], Pablo Merino[1,3,4], Abhishek Grewal[1], Christopher C. Leon[1], Klaus Kuhnke[1,*], Klaus Kern[1,5]

[1] Max-Planck-Institut für Festkörperforschung, D-70569, Stuttgart, Germany.

[2] Université de Strasbourg, CNRS, IPCMS, UMR 7504, F-67000 Strasbourg, France.

[3] Instituto de Ciencia de Materiales de Madrid, CSIC, E-28049, Madrid, Spain.

[4] Instituto de Física Fundamental, CSIC, E-28006, Madrid, Spain.

[5] Institut de Physique, École Polytechnique Fédérale de Lausanne, CH-1015 Lausanne, Switzerland.

* roslawska@ipcms.unistra.fr

* k.kuhnke@fkf.mpg.de

1. Single-atom deposition

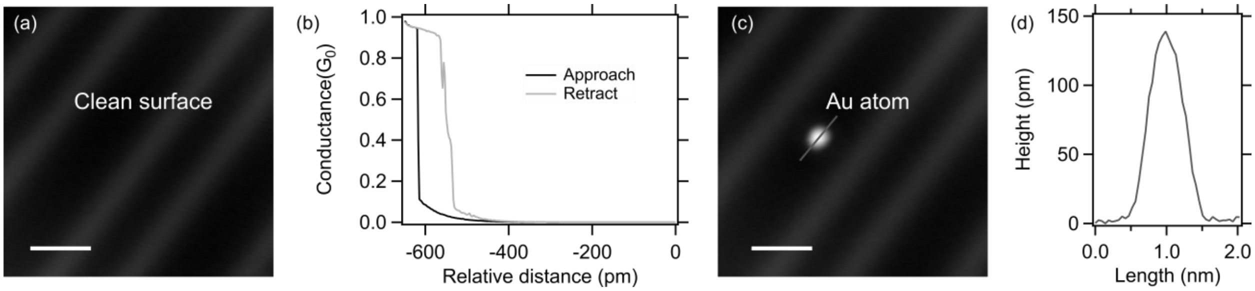

Figure S1. *Deposition of single Au atoms. a) Image of a clean Au(111) surface, I = 100 pA, U = 1 V. b) Approach and retract curve resulting in deposition of a single atom, U = 1 V, current set-point 100 pA. c) Scan of the same area as in a) after atom deposition. Scale bars 2 nm. d) Height profile showing a cross-section along the line indicated in (c).*

We prepare atomic-scale contacts used in our study by depositing individual atoms on the Au(111) surface. It can be controllably achieved by approaching the surface with the tip until contact[1] (Fig. S1). First, the surface is imaged to ensure it is clean, as presented in Fig. S1(a). Next, at a set-point of U = 1 V, I = 100 pA, the tip is moved towards the surface by 650 pm which results in a jump-to-contact event leading to a conductance close to 1 G_0 (Fig. S1(b)); the tip is subsequently retracted. Afterwards, the surface is imaged again to confirm successful atomic deposition(Fig. S1(c)). We find this procedure to be highly reproducible, which on occasion may also deposit a small cluster of a few atoms, which can be readily identified by a higher maximum conductance during the approach-retract curve and a higher topographic appearance than the one presented in Fig. S1(d). Since both the tip and the sample are made of Au, also the deposited structures consist of Au atoms.

2. Measurements with the feedback loop closed (Fig. 2)

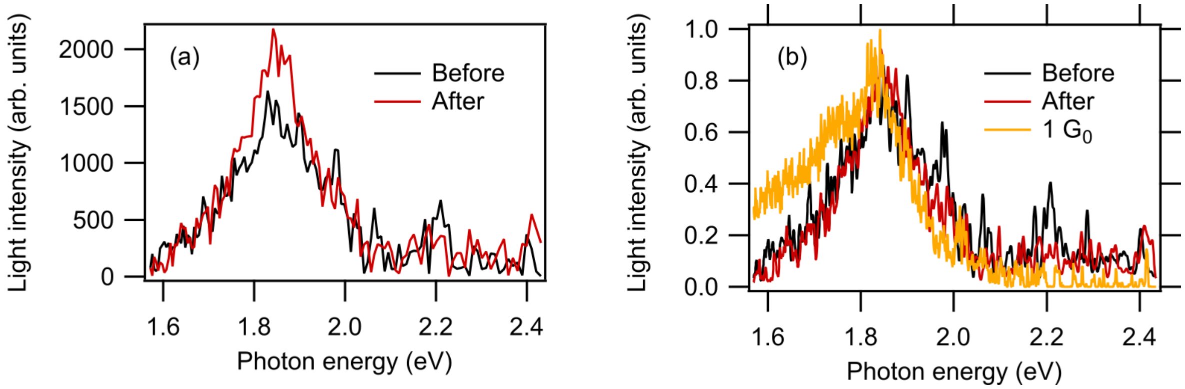

Figure S2. *(a) Optical spectra (binned) recorded on a clean Au(111) surface before and after measurements shown in Fig. 2 of the main manuscript, U = 3 V, I = 100 pA, integration time: 30 s. (b) Normalized reference spectra from (a) together with one of the spectra from Fig. 2(b), U = 1 V, I = 77.48 µA.*

Fig. S2 presents optical spectra recorded, before, during and after measurements presented in Fig. 2 of the main manuscript. We find that the shape of the spectrum did not change significantly after the measurement. The total integrated intensity, however, increased by 14 % (Fig. S2(a)). In Fig. S2(b) we compare these reference spectra in tunnel conditions (100 pA) with the spectrum recorded in contact (77 µA) and overbias emission condition. The main mode is slightly red-shifted with respect to the spectra recorded in tunneling. The overall higher intensity in the low energy regime (<1.8 eV) is a result of the overbias emission, which is less efficient at higher energies.

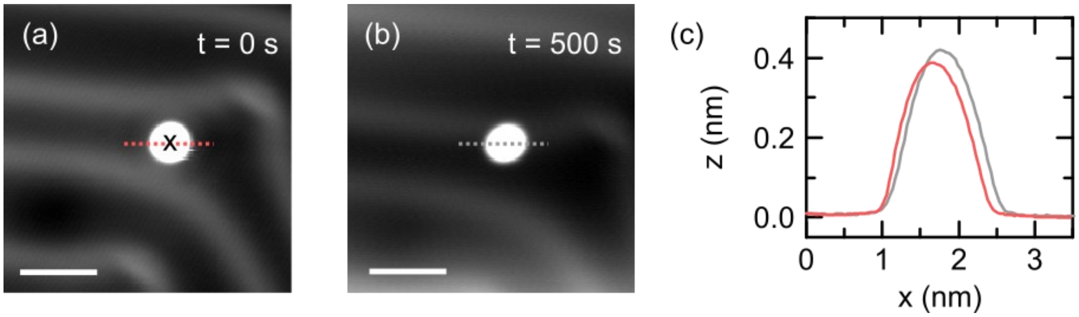

Figure S3. *(a), (b) STM images recorded before and after the contact measurement shown in Fig.2 of the main manuscript. The contact was formed at the marked position. U = 1 V, I = 100 pA, scale bars 3 nm. (c) Height profiles measured along the lines indicated in (a) and (b).*

Fig. S3 shows the area of the sample used to perform measurements presented in Fig. 2 of the main manuscript. The single-atom contact has been formed on top of the structure deposited from the tip as marked in Fig. S3(a). To evaluate the permanent modifications of the junction after the experiment, the surface was rescanned (Fig. S3(b)). We find that the investigated structure moved by 1 nm and likely an atom has been transferred to the surface, as indicated by the increased apparent height shown in Fig. S3(c). In this particular experiment, we contacted a larger adsorbed structure consisting of more than one atom because such junctions are more prone to spontaneous changes and thus more illustrative for our study. For the sake of completeness, we performed measurements in which we contacted a single atom deposited on the surface and observed both fluctuations when in contact and the permanent modification of electroluminescence as compared before and after the measurement. This experiment is described in the next section (Fig. S4 and Fig. S5).

3. Luminescence fluctuations in a point contact to a single atom deposited on the surface

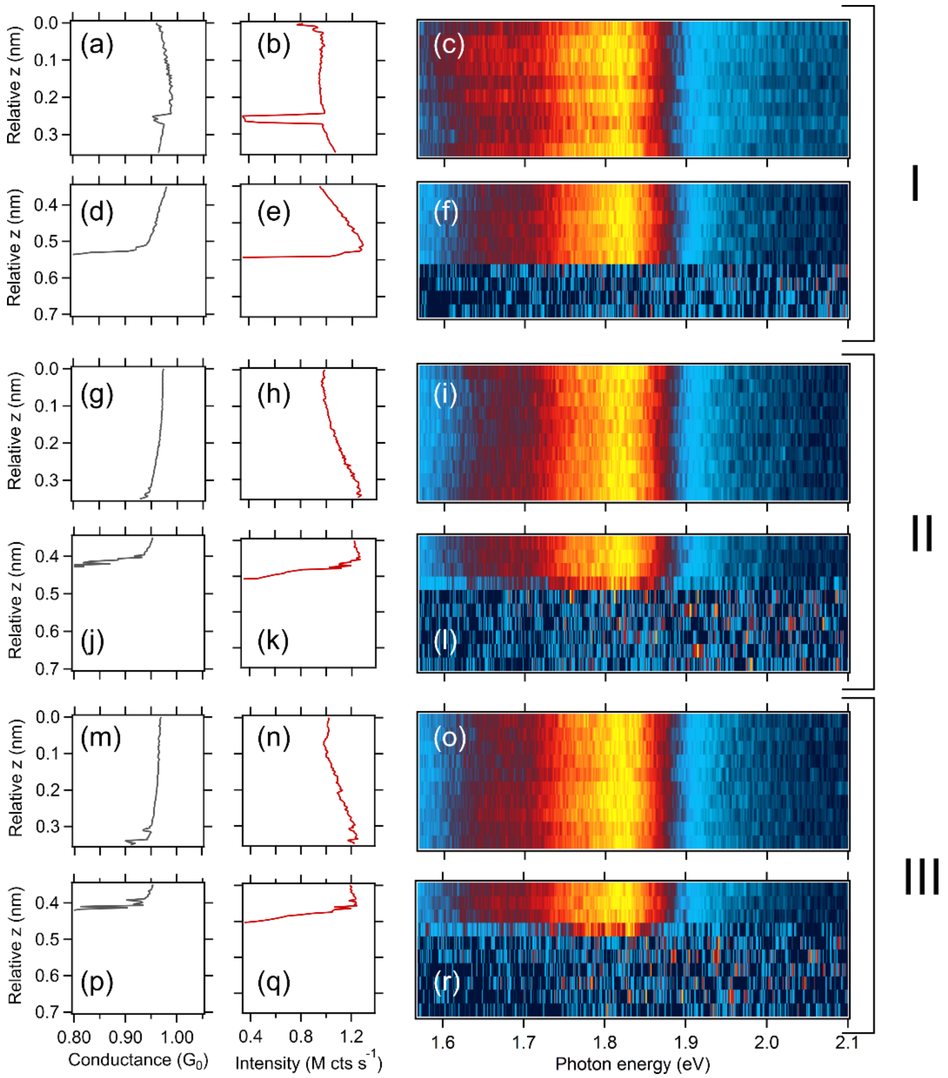

Figure S4. *Conductance and luminescence fluctuations during retraction from an atomic contact until rupture. The contact was formed on a single adatom as described earlier. The left column shows conductance traces, the middle column the spectrally integrated light intensity and, the right column emission spectra normalized to their maximum. Each plot in the right column consists of 10 spectra (5 s integration time). The whole figure presents 3 separate experiments (I, II, III), each recorded in two parts (for example a)-c) and d)-f)) accounting for the start of the stretching and the breaking of contact. In each part, the tip was retracted by 350 pm in steps of 3.5 pm. Between the separate experiments, the*

surface was inspected by a topographic scan and after that, the atomic contact was reestablished. Overbias emission condition U = 1 V.

In the experiments displayed in Fig. S4 we deposited a single Au atom (see Fig. S5), established a contact of 1 G_0, and then retracted the tip until loss of contact (rupture). Simultaneously, we monitored the electroluminescence integrated intensity (middle column in Fig. S4) and the overbias emission spectrum (right column in Fig. S4). No current feedback was used throughout the experiment. The measurements were performed in an extremely gentle fashion, each time the tip was continuously retracted (in steps of 3.5 pm) by 350 pm in total. If the contact did not break, the tip was retracted by another 350 pm. We found that typically the contact broke after a total retraction of approx. 450 pm. After each experiment, we rescanned the surface and repeated the experiment on the same atom following the same protocol. This procedure was reproducible and the approach-retract routine could be repeated on the same atom multiple times, as shown in Fig. S4. Similar to the measurements presented in Fig. 2 and Fig. 3 of the main text, the luminescence can evolve in both gradual and step-wise manner which can be correlated with the changes in the conductance (left column in Fig. S4). Again, the spectral shape remains unchanged as displayed in the right column of Fig. S4. The tip was stabilized for 15 h before the series of experiments was started.

Fig. S5 shows STM images recorded before (Fig. S5(a)) and after (Fig. S5(b)) accomplishing all experiments presented in Fig. S4, the investigated atom (deposited from the tip as described above) is marked by an arrow. We find that at the end of the experiments the deposited adatom had moved by 1 nm in total but no additional atom had been transferred, which demonstrates the reproducible experimental conditions. In contrast, the apex of the tip has evidently changed: First, the deposited atom and the defects on the surface are imaged as features with smaller extension in Fig. S5(b) than in Fig. S5(a) indicating that the tip apex has become sharper. Second, there is a decrease in the light intensity by 8 % percent (Fig. S5(c)).

These observations further confirm that a minute change at the tip structure, even without transferring an atom to the surface, may induce a modification in the plasmonic properties of the junction.

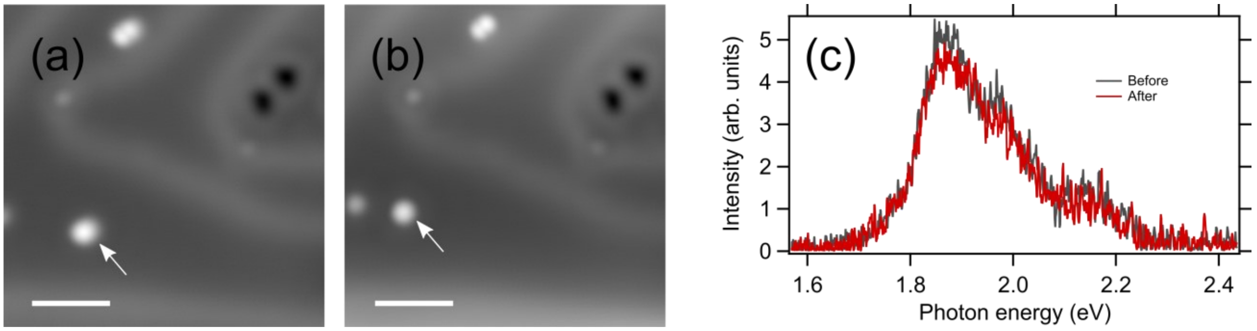

Figure S5. *Stability of a single atom on the surface during measurements in contact. (a), (b) STM images recorded before and after measurements presented in Fig. S4, which were performed on the atom marked by an arrow. U = 1 V, I = 100 pA, scale bars 2 nm. (c) Optical spectra recorded before and after the measurements presented in Fig. S4, tunnel conditions U = 3 V, I = 100 pA.*

4. Local heat dissipation in the junction

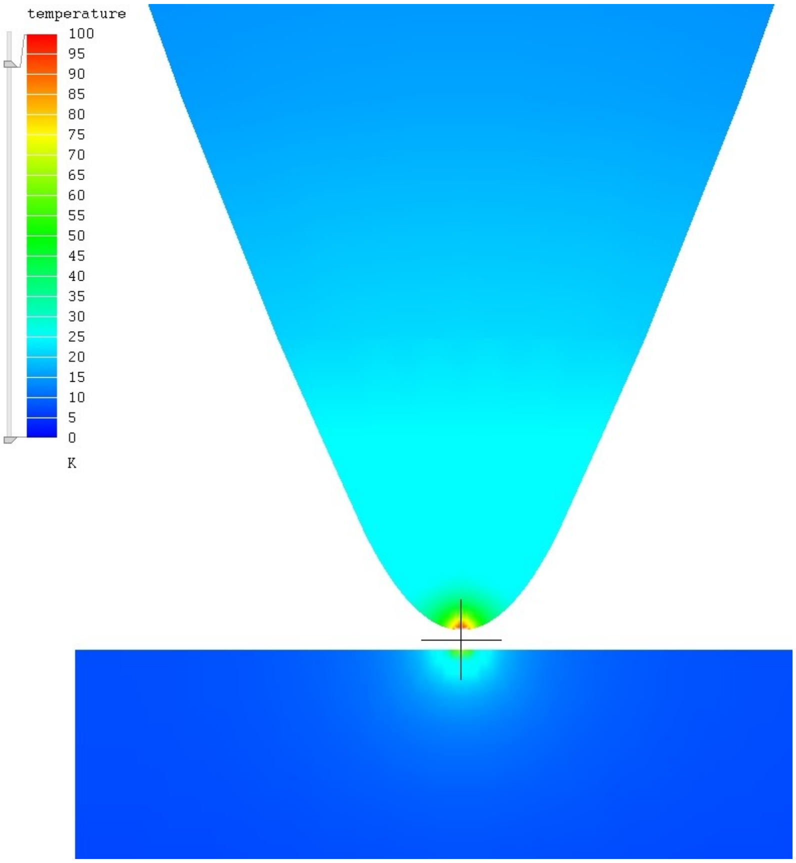

Figure S6. *Temperature distribution in a plane cut through the 3-dimensional geometry of the junction. The distribution was determined using the Mecway finite element software (Mecway Ltd., New Zealand). We used the temperature-dependent thermal conductivity reported for unannealed high purity (99.99%) Au wires[2]. Tip curvature radius ca. 3 nm, the opening angle of a cone ca. 45°. Length scale: The tip-sample distance is set to 1 nm exactly. A power of 40 µW (half of the dissipation at 1 G_0) is input over an area of ca 1 nm^2 at the contact point independently to both tip and sample. No further heat transfer between tip and sample is assumed.*

In Fig. 2e of the main text, we observe thermal tip elongation due to the power dissipated in the junction. Since the main part of the applied voltage drops across the tip-substrate contact region, we can estimate the dissipated power at the junction to be close to $U^2 G_0 \approx 77$ µW. About half of the power will be

dissipated on the tip and substrate each. However, since heat transport in the substrate occurs over a hemispherical (2π) region in the macroscopic Au crystal while the tip is sharp and conical, the strongest contribution to thermal expansion will be due to heat dissipation in the tip. Coarse modeling of heat flow at a conical gold tip suggests a temperature of the order of 30 K to 100 K (see Fig. S6) which may, however, be surpassed in close vicinity to the atomic junction. One can also define the upper temperature bound to be well below a significant fraction of the melting temperature of Au. If such high temperatures were to arise, we would observe a permanent instability of the tunnel junction due to rapid atomic diffusion on much faster time scales than actually observed (Fig. S3 and S5). The simulation demonstrates that the conical tip exhibits much higher temperatures due to the reduced effective dimensionality in comparison to the half-space available for heat transport in the substrate even when equal shares of the total dissipated electric power are fed to tip and sample.

In laser-based plasmon-enhanced spectroscopies (as e.g., TERS or TEPL) the thermal input by the illumination can become comparable to the power dissipation by the electric current and higher ambient temperatures will play an even greater role. While our study tries to limit heat input by using low voltages and cryogenic temperature, the thermal reorganization will be most relevant at higher conductance (4-5 G_0) since here the heat dissipation at the junction is increased by the respective prefactor.

5. Optical spectroscopy at high conductances

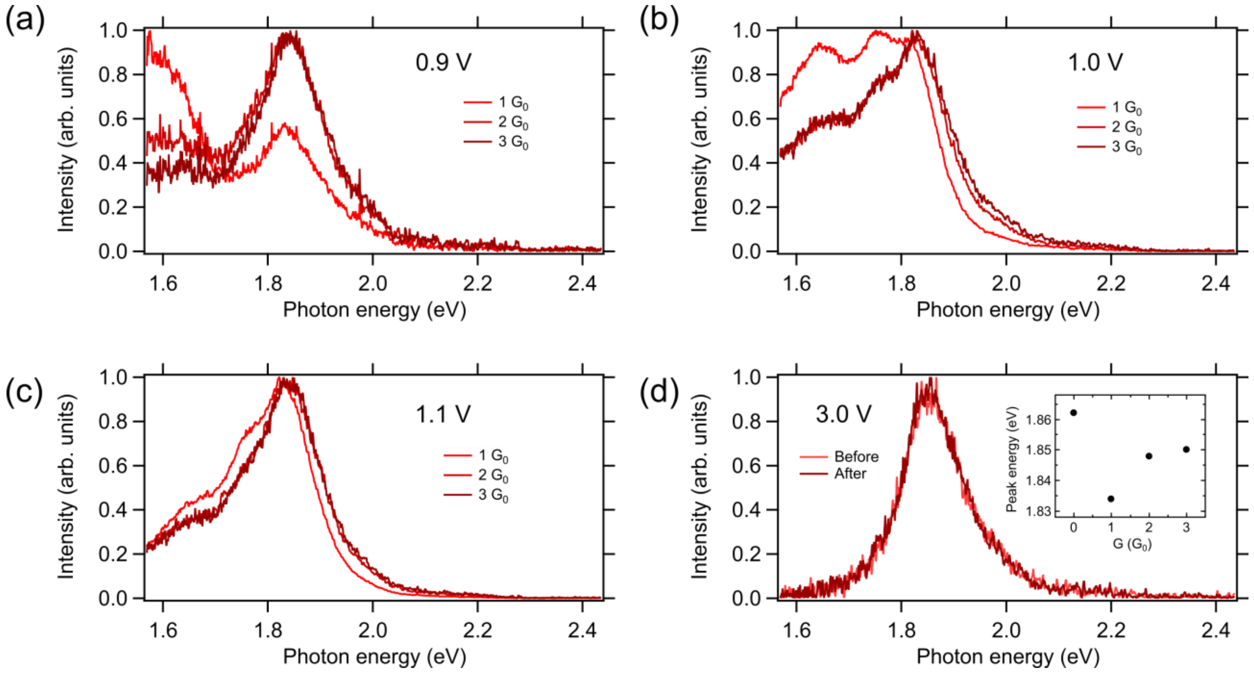

Figure S7. *Optical spectra as a function of conductance and bias. (a)-(c) Normalized optical overbias spectra as a function of bias and conductance, with the values indicated next to the traces. The current feedback was enabled during the measurements. (d) Normalized reference spectra recorded at tunneling conditions before and after the measurements presented in a)-c), U = 3 V, I = 100 pA (corresponding to $4*10^{-7}$ G_0). The inset shows the energies of the main plasmonic mode as a function of conductance, the values for measurements in contact are taken from data in (c).*

Fig. S7 presents a series of spectra recorded for different bias voltages and different conductances. Fig. S7(d) shows reference spectra recorded before and after the series to demonstrate that the plasmonic modes of the junction have not been altered by the measurement. The shapes of the spectra are strongly bias-dependent because of the strong non-linearity of the overbias emission. Remarkably, the relative intensity of the low-energy component is highest at 1 G_0. For each studied bias in Fig. S7a-c, the spectra for 2 and 3 G_0 nearly overlap with a well-pronounced maximum around 1.8 eV, whereas at 1 G_0 the

luminescence intensity at $h\nu < 1.8$ eV may be as high or even higher than the intensity of 1.8 eV maximum, as in Fig. S7b. This effect may be related to a reduced probability of electron-electron interaction[3] due to the limited number of participating transport channels under these conditions, which can be as low as 1. When the electrons pass mainly through one channel, one may expect the interaction between successive electrons to be reduced due to their spatiotemporal separation. We would like to note that the peak energy of the main mode shifts by about 30 mV. It is highest at tunneling conditions (1.86 eV), lowest (1.83 eV) at 1 G_0, and increases again with the conductance reaching 1.85 eV at 3 G_0 (see the inset in Fig. S7(d)). This is in qualitative agreement with the ab initio[4,5] and quantum plasmonic predictions[6].

6. Emission minimum around integer G_0

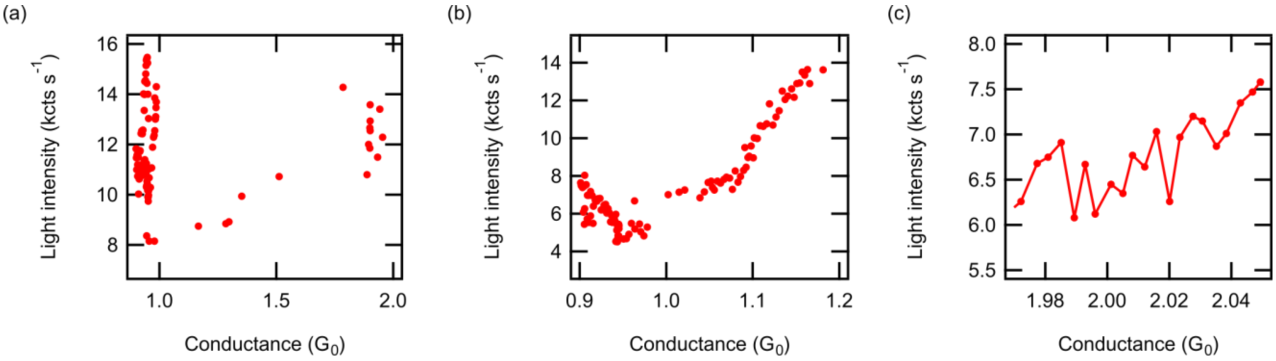

Figure S8*. Light intensity vs. conductance plots extracted from Fig. 3ab of the main text.*

Fig. S8a and Fig.8b show light intensity as a function of conductance plots extracted from Fig. 3a and Fig.3b of the main text, respectively. Similar to the plot presented in Fig. 3d of the main text, there is a minimum of light emission at values close to 1 G_0. In Fig. S8c we plot the light intensity extracted from Fig. 3b of the main text suggesting a shallow minimum at an integer conductance value of 2 G_0.

References

(1) Limot, L.; Kröger, J.; Berndt, R.; Garcia-Lekue, A.; Hofer, W. A. Atom Transfer and Single-Adatom Contacts. *Phys. Rev. Lett.* **2005**, *94* (12), 126102.

(2) White, G. K. The Thermal Conductivity of Gold at Low Temperatures. *Proc. Phys. Soc. Sect. A* **1953**, *66* (6), 559–564.

(3) Peters, P.-J.; Xu, F.; Kaasbjerg, K.; Rastelli, G.; Belzig, W.; Berndt, R. Quantum Coherent Multielectron Processes in an Atomic Scale Contact. *Phys. Rev. Lett.* **2017**, *119* (6), 066803.

(4) Rossi, T. P.; Zugarramurdi, A.; Puska, M. J.; Nieminen, R. M. Quantized Evolution of the Plasmonic Response in a Stretched Nanorod. *Phys. Rev. Lett.* **2015**, *115* (23), 236804.

(5) Marchesin, F.; Koval, P.; Barbry, M.; Aizpurua, J.; Sánchez-Portal, D. Plasmonic Response of Metallic Nanojunctions Driven by Single Atom Motion: Quantum Transport Revealed in Optics. *ACS Photonics* **2016**, *3* (2), 269–277.

(6) Esteban, R.; Borisov, A. G.; Nordlander, P.; Aizpurua, J. Bridging Quantum and Classical Plasmonics with a Quantum-Corrected Model. *Nat. Commun.* **2012**, *3*, 825.
